# *N*-(4-Chloro­benzo­yl)-2-methyl­benzene­sulfonamide

**DOI:** 10.1107/S1600536810026759

**Published:** 2010-07-14

**Authors:** P. A. Suchetan, B. Thimme Gowda, Sabine Foro, Hartmut Fuess

**Affiliations:** a Department of Chemistry, Mangalore University, Mangalagangotri 574 199, Mangalore, India; b Institute of Materials Science, Darmstadt University of Technology, Petersenstrasse 23, D-64287 Darmstadt, Germany

## Abstract

The asymmetric unit of the title compound, C_14_H_12_ClNO_3_S, contains two independent mol­ecules. The conformations of the N—C bonds in the C—SO_2_—NH—C(O) segments have *gauche* torsions with respect to the S=O bonds. The mol­ecules are twisted at the S atoms with torsion angles of −54.2 (2) and 63.8 (2)° in the two mol­ecules. The dihedral angles between the sulfonyl benzene rings and the —SO_2_—NH—C—O segments are 85.0 (1) and 87.0 (1)°. Furthermore, the dihedral angles between the sulfonyl and benzoyl benzene rings are 89.4 (1) and 82.4 (1)° in the two mol­ecules. In the crystal, mol­ecules are linked by N—H⋯O(S) hydrogen bonds.

## Related literature

For background literature and similar structures, see: Gowda *et al.* (2010 ▶); Suchetan *et al.* (2010*a* ▶,*b* ▶,*c* ▶). 
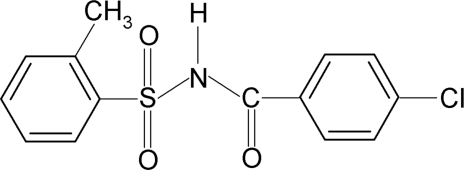


## Experimental

### 

#### Crystal data

C_14_H_12_ClNO_3_S
*M* *_r_* = 309.76
Triclinic, 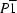

*a* = 10.9188 (9) Å
*b* = 12.157 (1) Å
*c* = 12.347 (1) Å
α = 60.533 (7)°
β = 84.705 (9)°
γ = 84.254 (9)°
*V* = 1418.0 (2) Å^3^
*Z* = 4
Mo *K*α radiation
μ = 0.42 mm^−1^
*T* = 299 K
0.38 × 0.24 × 0.14 mm

#### Data collection

Oxford Diffraction Xcalibur diffractometer with a Sapphire CCD detector
Absorption correction: multi-scan (*CrysAlis RED*; Oxford Diffraction, 2009 ▶) *T*_min_ = 0.856, *T*_max_ = 0.943
9975 measured reflections
5808 independent reflections
4476 reflections with *I* > 2σ(*I*)
*R*_int_ = 0.015

#### Refinement

*R*[*F* ^2^ > 2σ(*F* ^2^)] = 0.038
*wR*(*F* ^2^) = 0.110
*S* = 1.09
5808 reflections
369 parameters
2 restraints
H atoms treated by a mixture of independent and constrained refinement
Δρ_max_ = 0.32 e Å^−3^
Δρ_min_ = −0.37 e Å^−3^

### 

Data collection: *CrysAlis CCD* (Oxford Diffraction, 2009 ▶); cell refinement: *CrysAlis RED* (Oxford Diffraction, 2009 ▶); data reduction: *CrysAlis RED*; program(s) used to solve structure: *SHELXS97* (Sheldrick, 2008 ▶); program(s) used to refine structure: *SHELXL97* (Sheldrick, 2008 ▶); molecular graphics: *PLATON* (Spek, 2009 ▶); software used to prepare material for publication: *SHELXL97*.

## Supplementary Material

Crystal structure: contains datablocks I, global. DOI: 10.1107/S1600536810026759/ds2041sup1.cif

Structure factors: contains datablocks I. DOI: 10.1107/S1600536810026759/ds2041Isup2.hkl

Additional supplementary materials:  crystallographic information; 3D view; checkCIF report

## Figures and Tables

**Table 1:** Hydrogen-bond geometry (Å, °)

*D*—H⋯*A*	*D*—H	H⋯*A*	*D*⋯*A*	*D*—H⋯*A*
N1—H1*N*⋯O5^i^	0.82 (1)	2.13 (2)	2.937 (2)	168 (2)
N2—H2*N*⋯O1^i^	0.84 (2)	2.19 (2)	3.0195 (19)	171 (2)

Symmetry code: (i) 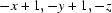.

**Fig. 1.**
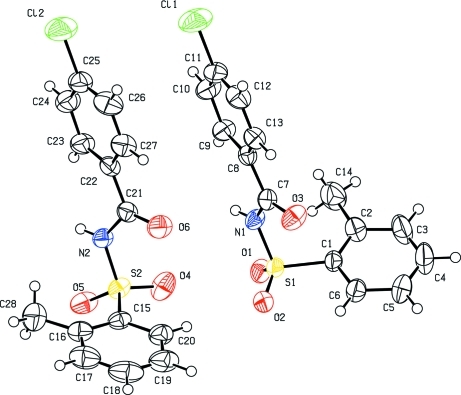
Molecular structure of the title compound, showing the atom-labelling scheme. Displacement ellipsoids are drawn at the 50% probability level.

**Fig. 2.**
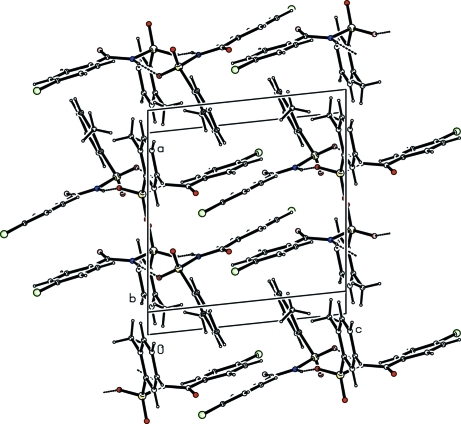
Molecular packing in the title compound. Hydrogen bonds are shown as dashed lines.

## supplementary crystallographic information

### Comment

Diaryl acylsulfonamides are known as potent antitumor agents against a broad spectrum of human tumor xenografts in nude mice. As a part of studying the effect of ring and the side chain substituents on the crystal structures of *N*-aromatic sulfonamides (Gowda *et al.*, 2010); Suchetan *et al.*, 2010*a,b*,*c*), the structure of 2-methyl-*N*-(4-chlorobenzoyl)benzenesulfonamide (I) has been determined. The asymmetric unit of the structure contains two independent molecules (Fig.1), similar to that observed in 2-methyl-*N*- (4-methylbenzoyl)benzenesulfonamide(II)(Gowda *et al.*, 2010*b*).

The conformations of the N—C bonds in the C—SO_2_—NH—C(O) segments have *gauche* torsions with respect to the SO bonds. Further, the conformations of the N—H bonds are *anti* to the C=O bonds, similar to those observed in (II), *N*-(4-chlorobenzoyl)benzenesulfonamide (Suchetan *et al.*, 2010*b*), 2-methyl-*N*-(benzoyl)- benzenesulfonamide (Suchetan *et al.*, 2010*c*) and 4-methyl-*N*-(4-chlorobenzoyl)benzenesulfonamide (V) (Suchetan *et al.*, 2010*a*).

The molecules are twisted at the the *S* atoms with the torsional angles of -54.2 (2)° and 63.8 (2)°, in the two independent molecules, compared to the values of -53.1 (2)° and 61.2 (2)° in the two molecules of (II). The dihedral angles between the sulfonyl benzene rings and the —SO_2_—NH—C—O segments are 85.0 (1)° and 87.0 (1)°, compared to the values of 86.0 (1)° (molecule 1) and 87.9 (1)° (molecule 2) in (II). Furthermore, the dihedral angles between the sulfonyl and the benzoyl benzene rings are 89.4 (1)° (molecule 1) and 82.1 (1)° (molecule 2), compared to the values of 88.1 (1)° (molecule 1) and 83.5 (1)° (molecule 2) in (II).

The packing of molecules linked by of N—H···O(S) hydrogen bonds (Table 1) is shown in Fig. 2.

### Experimental

The title compound was prepared by refluxing a mixture of 4-chlorobenzoic acid, 2-methylbenzenesulfonamide and phosphorous oxy chloride for 5 h on a water bath. The resultant mixture was cooled and poured into ice cold water. The solid, 2-methyl-*N*-(4-chlorobenzoyl)benzenesulfonamide obtained was filtered, washed thoroughly with water and then dissolved in sodium bicarbonate solution. The compound was later reprecipitated by acidifying the filtered solution with dilute HCl. The filtered and dried compound was recrystallized to the constant melting point.

Plate like colourless single crystals of the title compound used in X-ray diffraction studies were grown from a slow evaporation of its toluene solution at room temperature.

### Refinement

The H atoms of the NH groups were located in a difference map and later restrained to N—H = 0.86 (2) %A. The other H atoms were positioned with idealized geometry using a riding model with C—H = 0.93–0.96 Å. All H atoms were refined with isotropic displacement parameters (set to 1.2 times of the *U*_eq_ of the parent atom).

### Crystal data

**Table:**

C_14_H_12_ClNO_3_S	*Z* = 4
*M**_r_* = 309.76	*F*(000) = 640
Triclinic, *P*1	*D*_x_ = 1.451 Mg m^−^^3^
Hall symbol: -P 1	Mo *K*α radiation, λ = 0.71073 Å
*a* = 10.9188 (9) Å	Cell parameters from 4120 reflections
*b* = 12.157 (1) Å	θ = 2.5–27.8°
*c* = 12.347 (1) Å	µ = 0.42 mm^−^^1^
α = 60.533 (7)°	*T* = 299 K
β = 84.705 (9)°	Plate, colourless
γ = 84.254 (9)°	0.38 × 0.24 × 0.14 mm
*V* = 1418.0 (2) Å^3^	

### Data collection

**Table:**

Oxford Diffraction Xcalibur diffractometer with a Sapphire CCD detector	5808 independent reflections
Radiation source: fine-focus sealed tube	4476 reflections with *I* > 2σ(*I*)
graphite	*R*_int_ = 0.015
Rotation method data acquisition using ω and phi scans	θ_max_ = 26.4°, θ_min_ = 2.5°
Absorption correction: multi-scan (*CrysAlis RED*; Oxford Diffraction, 2009)	*h* = −12→13
*T*_min_ = 0.856, *T*_max_ = 0.943	*k* = −14→15
9975 measured reflections	*l* = −15→15

### Refinement

**Table:**

Refinement on *F*^2^	Primary atom site location: structure-invariant direct methods
Least-squares matrix: full	Secondary atom site location: difference Fourier map
*R*[*F*^2^ > 2σ(*F*^2^)] = 0.038	Hydrogen site location: inferred from neighbouring sites
*wR*(*F*^2^) = 0.110	H atoms treated by a mixture of independent and constrained refinement
*S* = 1.09	*w* = 1/[σ^2^(*F*_o_^2^) + (0.0583*P*)^2^ + 0.2188*P*] where *P* = (*F*_o_^2^ + 2*F*_c_^2^)/3
5808 reflections	(Δ/σ)_max_ = 0.014
369 parameters	Δρ_max_ = 0.32 e Å^−^^3^
2 restraints	Δρ_min_ = −0.37 e Å^−^^3^

### Special details

**Table:**

Experimental. (CrysAlis RED; Oxford Diffraction, 2009) Empirical absorption correction using spherical harmonics, implemented in SCALE3 ABSPACK scaling algorithm.
Geometry. All e.s.d.'s (except the e.s.d. in the dihedral angle between two l.s. planes) are estimated using the full covariance matrix. The cell e.s.d.'s are taken into account individually in the estimation of e.s.d.'s in distances, angles and torsion angles; correlations between e.s.d.'s in cell parameters are only used when they are defined by crystal symmetry. An approximate (isotropic) treatment of cell e.s.d.'s is used for estimating e.s.d.'s involving l.s. planes.
Refinement. Refinement of *F*^2^ against ALL reflections. The weighted *R*-factor *wR* and goodness of fit *S* are based on *F*^2^, conventional *R*-factors *R* are based on *F*, with *F* set to zero for negative *F*^2^. The threshold expression of *F*^2^ > σ(*F*^2^) is used only for calculating *R*-factors(gt) *etc*. and is not relevant to the choice of reflections for refinement. *R*-factors based on *F*^2^ are statistically about twice as large as those based on *F*, and *R*- factors based on ALL data will be even larger.

### Fractional atomic coordinates and isotropic or equivalent isotropic displacement parameters (Å^2^)

**Table:**

	*x*	*y*	*z*	*U*_iso_*/*U*_eq_	
Cl1	0.53923 (8)	−0.01400 (6)	0.72546 (6)	0.0865 (2)	
S1	0.23682 (4)	0.66981 (4)	0.15634 (4)	0.03693 (13)	
O1	0.20430 (13)	0.64973 (13)	0.05769 (12)	0.0483 (3)	
O2	0.31448 (13)	0.76921 (13)	0.12668 (14)	0.0525 (4)	
O3	0.30465 (14)	0.58139 (14)	0.40938 (13)	0.0522 (4)	
N1	0.30122 (15)	0.53209 (14)	0.25605 (14)	0.0373 (3)	
H1N	0.3207 (18)	0.4863 (18)	0.2247 (18)	0.045*	
C1	0.10164 (16)	0.69177 (17)	0.23579 (16)	0.0378 (4)	
C2	0.00714 (19)	0.6087 (2)	0.2801 (2)	0.0513 (5)	
C3	−0.0959 (2)	0.6412 (3)	0.3366 (2)	0.0692 (7)	
H3	−0.1613	0.5886	0.3669	0.083*	
C4	−0.1045 (2)	0.7472 (3)	0.3491 (3)	0.0737 (8)	
H4	−0.1750	0.7657	0.3871	0.088*	
C5	−0.0097 (2)	0.8268 (2)	0.3060 (2)	0.0618 (6)	
H5	−0.0154	0.8987	0.3152	0.074*	
C6	0.09419 (19)	0.7994 (2)	0.24898 (19)	0.0471 (5)	
H6	0.1590	0.8527	0.2195	0.056*	
C7	0.32853 (16)	0.50361 (17)	0.37495 (17)	0.0357 (4)	
C8	0.38324 (16)	0.37429 (17)	0.45661 (16)	0.0348 (4)	
C9	0.38889 (19)	0.27606 (18)	0.42869 (17)	0.0440 (5)	
H9	0.3609	0.2909	0.3535	0.053*	
C10	0.4358 (2)	0.1569 (2)	0.51173 (19)	0.0533 (5)	
H10	0.4382	0.0909	0.4936	0.064*	
C11	0.4790 (2)	0.1366 (2)	0.62180 (18)	0.0505 (5)	
C12	0.47585 (19)	0.2323 (2)	0.65098 (18)	0.0505 (5)	
H12	0.5066	0.2175	0.7251	0.061*	
C13	0.42662 (18)	0.35041 (19)	0.56893 (17)	0.0417 (4)	
H13	0.4223	0.4152	0.5889	0.050*	
C14	0.0106 (3)	0.4896 (3)	0.2710 (3)	0.0750 (8)	
H14A	0.0679	0.4274	0.3281	0.090*	
H14B	0.0362	0.5078	0.1877	0.090*	
H14C	−0.0700	0.4575	0.2915	0.090*	
Cl2	0.85328 (7)	−0.08027 (7)	0.55917 (7)	0.0865 (2)	
S2	0.62883 (5)	0.62957 (4)	−0.02845 (4)	0.03972 (13)	
O4	0.50003 (13)	0.64573 (15)	−0.00526 (16)	0.0597 (4)	
O5	0.67100 (15)	0.63364 (13)	−0.14372 (12)	0.0542 (4)	
O6	0.61855 (16)	0.51670 (14)	0.24179 (13)	0.0603 (4)	
N2	0.68281 (15)	0.48852 (14)	0.07786 (14)	0.0375 (4)	
H2N	0.7126 (18)	0.4425 (18)	0.0476 (18)	0.045*	
C15	0.70542 (18)	0.73952 (17)	−0.00952 (16)	0.0384 (4)	
C16	0.8319 (2)	0.75339 (19)	−0.03724 (19)	0.0491 (5)	
C17	0.8810 (3)	0.8448 (2)	−0.0200 (2)	0.0676 (7)	
H17	0.9649	0.8566	−0.0363	0.081*	
C18	0.8085 (3)	0.9179 (2)	0.0206 (2)	0.0729 (8)	
H18	0.8442	0.9783	0.0307	0.087*	
C19	0.6851 (3)	0.9033 (2)	0.0461 (2)	0.0646 (7)	
H19	0.6369	0.9539	0.0727	0.077*	
C20	0.6324 (2)	0.81276 (19)	0.03204 (19)	0.0490 (5)	
H20	0.5486	0.8010	0.0503	0.059*	
C21	0.66464 (18)	0.44628 (18)	0.20434 (17)	0.0388 (4)	
C22	0.71053 (17)	0.31356 (17)	0.28858 (17)	0.0382 (4)	
C23	0.7281 (2)	0.22033 (19)	0.25341 (19)	0.0502 (5)	
H23	0.7112	0.2396	0.1733	0.060*	
C24	0.7706 (2)	0.0992 (2)	0.3372 (2)	0.0589 (6)	
H24	0.7814	0.0365	0.3141	0.071*	
C25	0.7969 (2)	0.0719 (2)	0.4549 (2)	0.0535 (5)	
C26	0.7777 (2)	0.1624 (2)	0.4918 (2)	0.0599 (6)	
H26	0.7946	0.1424	0.5721	0.072*	
C27	0.7332 (2)	0.2826 (2)	0.40918 (19)	0.0506 (5)	
H27	0.7182	0.3434	0.4345	0.061*	
C28	0.9158 (2)	0.6772 (3)	−0.0839 (3)	0.0725 (7)	
H28A	0.8994	0.5893	−0.0350	0.087*	
H28B	1.0002	0.6877	−0.0768	0.087*	
H28C	0.9012	0.7063	−0.1696	0.087*	

### Atomic displacement parameters (Å^2^)

**Table:**

	*U*^11^	*U*^22^	*U*^33^	*U*^12^	*U*^13^	*U*^23^
Cl1	0.1194 (6)	0.0570 (4)	0.0559 (4)	0.0228 (4)	−0.0283 (4)	−0.0077 (3)
S1	0.0389 (3)	0.0364 (2)	0.0317 (2)	0.00455 (18)	0.00168 (17)	−0.01557 (19)
O1	0.0541 (9)	0.0564 (9)	0.0314 (7)	0.0137 (7)	−0.0049 (6)	−0.0217 (6)
O2	0.0472 (8)	0.0416 (8)	0.0613 (9)	−0.0045 (6)	0.0141 (7)	−0.0218 (7)
O3	0.0675 (10)	0.0513 (9)	0.0521 (9)	0.0094 (7)	−0.0155 (7)	−0.0365 (7)
N1	0.0461 (9)	0.0364 (8)	0.0328 (8)	0.0075 (7)	−0.0056 (6)	−0.0207 (7)
C1	0.0356 (10)	0.0425 (10)	0.0336 (9)	0.0042 (8)	0.0009 (7)	−0.0188 (8)
C2	0.0475 (12)	0.0581 (13)	0.0498 (12)	−0.0063 (10)	0.0060 (9)	−0.0283 (11)
C3	0.0473 (14)	0.0861 (19)	0.0768 (17)	−0.0161 (13)	0.0226 (12)	−0.0437 (15)
C4	0.0531 (15)	0.094 (2)	0.0835 (19)	0.0027 (14)	0.0197 (13)	−0.0554 (17)
C5	0.0569 (14)	0.0690 (16)	0.0717 (16)	0.0097 (12)	0.0038 (12)	−0.0474 (14)
C6	0.0444 (11)	0.0486 (12)	0.0504 (12)	0.0027 (9)	0.0010 (9)	−0.0274 (10)
C7	0.0365 (10)	0.0413 (10)	0.0361 (9)	−0.0020 (8)	−0.0026 (7)	−0.0240 (8)
C8	0.0357 (9)	0.0392 (10)	0.0304 (9)	−0.0024 (7)	0.0000 (7)	−0.0179 (8)
C9	0.0604 (13)	0.0429 (11)	0.0298 (9)	0.0047 (9)	−0.0080 (8)	−0.0190 (9)
C10	0.0759 (16)	0.0412 (11)	0.0418 (11)	0.0081 (10)	−0.0082 (10)	−0.0206 (9)
C11	0.0587 (13)	0.0463 (12)	0.0335 (10)	0.0056 (10)	−0.0066 (9)	−0.0102 (9)
C12	0.0549 (13)	0.0611 (13)	0.0312 (10)	−0.0055 (10)	−0.0077 (9)	−0.0179 (10)
C13	0.0459 (11)	0.0487 (11)	0.0348 (10)	−0.0066 (9)	−0.0017 (8)	−0.0230 (9)
C14	0.0712 (17)	0.0717 (17)	0.093 (2)	−0.0250 (14)	0.0215 (15)	−0.0486 (16)
Cl2	0.0881 (5)	0.0596 (4)	0.0751 (5)	0.0108 (3)	−0.0196 (4)	−0.0047 (3)
S2	0.0506 (3)	0.0371 (3)	0.0370 (3)	0.0045 (2)	−0.0112 (2)	−0.0221 (2)
O4	0.0473 (9)	0.0643 (10)	0.0845 (11)	0.0069 (7)	−0.0177 (8)	−0.0489 (9)
O5	0.0895 (12)	0.0437 (8)	0.0333 (7)	0.0062 (7)	−0.0142 (7)	−0.0218 (6)
O6	0.0961 (12)	0.0468 (9)	0.0420 (8)	0.0014 (8)	0.0093 (8)	−0.0278 (7)
N2	0.0517 (10)	0.0327 (8)	0.0314 (8)	0.0000 (7)	−0.0002 (7)	−0.0190 (7)
C15	0.0542 (12)	0.0303 (9)	0.0294 (9)	0.0001 (8)	−0.0093 (8)	−0.0130 (8)
C16	0.0551 (13)	0.0415 (11)	0.0432 (11)	−0.0033 (9)	−0.0086 (9)	−0.0139 (9)
C17	0.0730 (17)	0.0552 (14)	0.0662 (16)	−0.0197 (12)	−0.0104 (12)	−0.0193 (13)
C18	0.116 (2)	0.0456 (14)	0.0595 (15)	−0.0268 (15)	−0.0114 (15)	−0.0223 (12)
C19	0.107 (2)	0.0414 (12)	0.0523 (13)	−0.0113 (13)	0.0004 (13)	−0.0275 (11)
C20	0.0669 (14)	0.0397 (11)	0.0411 (11)	−0.0020 (9)	−0.0023 (9)	−0.0206 (9)
C21	0.0483 (11)	0.0390 (10)	0.0339 (9)	−0.0080 (8)	0.0036 (8)	−0.0214 (8)
C22	0.0433 (11)	0.0386 (10)	0.0328 (9)	−0.0092 (8)	0.0045 (8)	−0.0175 (8)
C23	0.0733 (15)	0.0413 (11)	0.0341 (10)	−0.0016 (10)	0.0005 (9)	−0.0179 (9)
C24	0.0786 (17)	0.0448 (12)	0.0494 (13)	0.0030 (11)	0.0024 (11)	−0.0221 (10)
C25	0.0501 (13)	0.0458 (12)	0.0489 (12)	−0.0008 (10)	−0.0045 (9)	−0.0112 (10)
C26	0.0701 (16)	0.0637 (15)	0.0389 (11)	−0.0127 (12)	−0.0121 (10)	−0.0166 (11)
C27	0.0650 (14)	0.0499 (12)	0.0412 (11)	−0.0098 (10)	−0.0029 (10)	−0.0245 (10)
C28	0.0545 (15)	0.0732 (18)	0.0840 (19)	0.0025 (13)	0.0013 (13)	−0.0359 (15)

### Geometric parameters (Å, °)

**Table:**

Cl1—C11	1.741 (2)	Cl2—C25	1.742 (2)
S1—O2	1.4234 (15)	S2—O4	1.4227 (15)
S1—O1	1.4350 (14)	S2—O5	1.4333 (14)
S1—N1	1.6460 (16)	S2—N2	1.6543 (16)
S1—C1	1.7674 (17)	S2—C15	1.7629 (19)
O3—C7	1.212 (2)	O6—C21	1.208 (2)
N1—C7	1.387 (2)	N2—C21	1.384 (2)
N1—H1N	0.823 (14)	N2—H2N	0.840 (15)
C1—C6	1.389 (3)	C15—C20	1.387 (3)
C1—C2	1.395 (3)	C15—C16	1.398 (3)
C2—C3	1.396 (3)	C16—C17	1.393 (3)
C2—C14	1.502 (3)	C16—C28	1.512 (3)
C3—C4	1.365 (4)	C17—C18	1.375 (4)
C3—H3	0.9300	C17—H17	0.9300
C4—C5	1.372 (3)	C18—C19	1.364 (4)
C4—H4	0.9300	C18—H18	0.9300
C5—C6	1.379 (3)	C19—C20	1.381 (3)
C5—H5	0.9300	C19—H19	0.9300
C6—H6	0.9300	C20—H20	0.9300
C7—C8	1.486 (3)	C21—C22	1.490 (3)
C8—C13	1.389 (2)	C22—C27	1.385 (3)
C8—C9	1.391 (3)	C22—C23	1.391 (3)
C9—C10	1.378 (3)	C23—C24	1.381 (3)
C9—H9	0.9300	C23—H23	0.9300
C10—C11	1.377 (3)	C24—C25	1.375 (3)
C10—H10	0.9300	C24—H24	0.9300
C11—C12	1.374 (3)	C25—C26	1.375 (3)
C12—C13	1.375 (3)	C26—C27	1.377 (3)
C12—H12	0.9300	C26—H26	0.9300
C13—H13	0.9300	C27—H27	0.9300
C14—H14A	0.9600	C28—H28A	0.9600
C14—H14B	0.9600	C28—H28B	0.9600
C14—H14C	0.9600	C28—H28C	0.9600
			
O2—S1—O1	118.58 (9)	O4—S2—O5	118.63 (9)
O2—S1—N1	110.72 (9)	O4—S2—N2	110.03 (9)
O1—S1—N1	103.77 (8)	O5—S2—N2	103.34 (8)
O2—S1—C1	108.12 (9)	O4—S2—C15	108.98 (9)
O1—S1—C1	109.66 (9)	O5—S2—C15	109.55 (9)
N1—S1—C1	105.17 (8)	N2—S2—C15	105.45 (8)
C7—N1—S1	122.68 (12)	C21—N2—S2	122.47 (13)
C7—N1—H1N	124.9 (15)	C21—N2—H2N	123.9 (14)
S1—N1—H1N	112.2 (15)	S2—N2—H2N	113.1 (14)
C6—C1—C2	121.90 (18)	C20—C15—C16	122.23 (18)
C6—C1—S1	115.49 (15)	C20—C15—S2	116.02 (16)
C2—C1—S1	122.59 (15)	C16—C15—S2	121.74 (15)
C1—C2—C3	116.0 (2)	C17—C16—C15	116.1 (2)
C1—C2—C14	124.48 (19)	C17—C16—C28	119.3 (2)
C3—C2—C14	119.5 (2)	C15—C16—C28	124.58 (19)
C4—C3—C2	122.5 (2)	C18—C17—C16	121.7 (2)
C4—C3—H3	118.7	C18—C17—H17	119.2
C2—C3—H3	118.7	C16—C17—H17	119.2
C3—C4—C5	120.4 (2)	C19—C18—C17	121.1 (2)
C3—C4—H4	119.8	C19—C18—H18	119.4
C5—C4—H4	119.8	C17—C18—H18	119.4
C4—C5—C6	119.5 (2)	C18—C19—C20	119.4 (2)
C4—C5—H5	120.3	C18—C19—H19	120.3
C6—C5—H5	120.3	C20—C19—H19	120.3
C5—C6—C1	119.7 (2)	C19—C20—C15	119.4 (2)
C5—C6—H6	120.1	C19—C20—H20	120.3
C1—C6—H6	120.1	C15—C20—H20	120.3
O3—C7—N1	120.10 (17)	O6—C21—N2	120.58 (18)
O3—C7—C8	122.85 (16)	O6—C21—C22	123.14 (16)
N1—C7—C8	117.02 (14)	N2—C21—C22	116.21 (15)
C13—C8—C9	118.88 (17)	C27—C22—C23	119.24 (19)
C13—C8—C7	117.39 (16)	C27—C22—C21	117.08 (17)
C9—C8—C7	123.69 (16)	C23—C22—C21	123.65 (17)
C10—C9—C8	120.43 (18)	C24—C23—C22	120.20 (19)
C10—C9—H9	119.8	C24—C23—H23	119.9
C8—C9—H9	119.8	C22—C23—H23	119.9
C11—C10—C9	119.27 (19)	C25—C24—C23	119.5 (2)
C11—C10—H10	120.4	C25—C24—H24	120.3
C9—C10—H10	120.4	C23—C24—H24	120.3
C12—C11—C10	121.47 (19)	C24—C25—C26	120.9 (2)
C12—C11—Cl1	119.62 (16)	C24—C25—Cl2	119.30 (18)
C10—C11—Cl1	118.90 (17)	C26—C25—Cl2	119.76 (18)
C11—C12—C13	119.00 (18)	C25—C26—C27	119.7 (2)
C11—C12—H12	120.5	C25—C26—H26	120.2
C13—C12—H12	120.5	C27—C26—H26	120.2
C12—C13—C8	120.93 (18)	C26—C27—C22	120.4 (2)
C12—C13—H13	119.5	C26—C27—H27	119.8
C8—C13—H13	119.5	C22—C27—H27	119.8
C2—C14—H14A	109.5	C16—C28—H28A	109.5
C2—C14—H14B	109.5	C16—C28—H28B	109.5
H14A—C14—H14B	109.5	H28A—C28—H28B	109.5
C2—C14—H14C	109.5	C16—C28—H28C	109.5
H14A—C14—H14C	109.5	H28A—C28—H28C	109.5
H14B—C14—H14C	109.5	H28B—C28—H28C	109.5
			
O2—S1—N1—C7	62.39 (16)	O4—S2—N2—C21	−53.60 (17)
O1—S1—N1—C7	−169.35 (14)	O5—S2—N2—C21	178.78 (15)
C1—S1—N1—C7	−54.17 (17)	C15—S2—N2—C21	63.79 (17)
O2—S1—C1—C6	2.61 (18)	O4—S2—C15—C20	4.60 (18)
O1—S1—C1—C6	−128.04 (15)	O5—S2—C15—C20	135.89 (15)
N1—S1—C1—C6	120.93 (16)	N2—S2—C15—C20	−113.49 (15)
O2—S1—C1—C2	−178.77 (17)	O4—S2—C15—C16	−174.18 (15)
O1—S1—C1—C2	50.57 (19)	O5—S2—C15—C16	−42.90 (18)
N1—S1—C1—C2	−60.45 (18)	N2—S2—C15—C16	67.72 (17)
C6—C1—C2—C3	1.0 (3)	C20—C15—C16—C17	0.4 (3)
S1—C1—C2—C3	−177.57 (18)	S2—C15—C16—C17	179.11 (16)
C6—C1—C2—C14	−178.7 (2)	C20—C15—C16—C28	−179.4 (2)
S1—C1—C2—C14	2.7 (3)	S2—C15—C16—C28	−0.7 (3)
C1—C2—C3—C4	−0.5 (4)	C15—C16—C17—C18	−0.8 (3)
C14—C2—C3—C4	179.3 (3)	C28—C16—C17—C18	179.1 (2)
C2—C3—C4—C5	−0.2 (5)	C16—C17—C18—C19	0.3 (4)
C3—C4—C5—C6	0.5 (4)	C17—C18—C19—C20	0.6 (4)
C4—C5—C6—C1	0.0 (4)	C18—C19—C20—C15	−1.0 (3)
C2—C1—C6—C5	−0.8 (3)	C16—C15—C20—C19	0.4 (3)
S1—C1—C6—C5	177.85 (17)	S2—C15—C20—C19	−178.33 (16)
S1—N1—C7—O3	0.2 (3)	S2—N2—C21—O6	−7.4 (3)
S1—N1—C7—C8	178.24 (12)	S2—N2—C21—C22	175.53 (13)
O3—C7—C8—C13	−10.8 (3)	O6—C21—C22—C27	−18.8 (3)
N1—C7—C8—C13	171.28 (16)	N2—C21—C22—C27	158.22 (18)
O3—C7—C8—C9	166.76 (19)	O6—C21—C22—C23	159.5 (2)
N1—C7—C8—C9	−11.2 (3)	N2—C21—C22—C23	−23.6 (3)
C13—C8—C9—C10	0.6 (3)	C27—C22—C23—C24	−1.6 (3)
C7—C8—C9—C10	−176.92 (18)	C21—C22—C23—C24	−179.78 (19)
C8—C9—C10—C11	−1.1 (3)	C22—C23—C24—C25	−0.9 (3)
C9—C10—C11—C12	0.3 (3)	C23—C24—C25—C26	2.2 (3)
C9—C10—C11—Cl1	−179.44 (17)	C23—C24—C25—Cl2	−178.80 (17)
C10—C11—C12—C13	1.0 (3)	C24—C25—C26—C27	−1.0 (3)
Cl1—C11—C12—C13	−179.22 (15)	Cl2—C25—C26—C27	179.98 (17)
C11—C12—C13—C8	−1.6 (3)	C25—C26—C27—C22	−1.5 (3)
C9—C8—C13—C12	0.8 (3)	C23—C22—C27—C26	2.8 (3)
C7—C8—C13—C12	178.45 (17)	C21—C22—C27—C26	−178.91 (19)

### Hydrogen-bond geometry (Å, °)

**Table:**

*D*—H···*A*	*D*—H	H···*A*	*D*···*A*	*D*—H···*A*
N1—H1N···O5^i^	0.82 (1)	2.13 (2)	2.937 (2)	168 (2)
N2—H2N···O1^i^	0.84 (2)	2.19 (2)	3.0195 (19)	171 (2)

Symmetry codes: (i) −*x*+1, −*y*+1, −*z*.
